# A Short Form of the Chinese Version of the Weinstein Noise Sensitivity Scale through Optimal Test Assembly

**DOI:** 10.3390/ijerph18030879

**Published:** 2021-01-20

**Authors:** Sha Li, Daniel Yee Tak Fong, Sarah Lai Yin Wan, Bradley McPherson, Esther Yuet Ying Lau, Lixi Huang, Mary Sau Man Ip, Janet Yuen Ha Wong

**Affiliations:** 1 School of Nursing, The University of Hong Kong, Hong Kong 999077, China; joylisha@connect.hku.hk (S.L.); janetyh@hku.hk (J.Y.H.W.); 2 Department of Psychology, The Education University of Hong Kong, Hong Kong 999077, China; sarahwan@eduhk.hk; 3 Division of Speech and Hearing Sciences, Faculty of Education, The University of Hong Kong, Hong Kong 999077, China; dbmcpher@hku.hk; 4 Sleep Laboratory, Department of Psychology, The Education University of Hong Kong, Hong Kong 999077, China; laueyy@eduhk.hk; 5 Centre for Psychosocial Health, The Education University of Hong Kong, Hong Kong 999077, China; 6 Department of Mechanical Engineering, The University of Hong Kong, Hong Kong 999077, China; lixi@hku.hk; 7 Department of Medicine, Li Ka Shing Faculty of Medicine, The University of Hong Kong, Hong Kong 999077, China; msmip@hku.hk

**Keywords:** item response theory, item selection, noise sensitivity, optimal test assembly, reliability, validity

## Abstract

This study developed a short form of the traditional Chinese version of the Weinstein Noise Sensitivity Scale (WNSS) through optimal test assembly (OTA). A total of 1069 Chinese adults (64.8% female) completed the territory-wide cross-sectional study. We first removed Items 12 and 5 which had negative factor loading and gender-related differential item functioning (DIF), respectively. The optimal length was then determined as the minimal one that reasonably resembled the reliability and validity of the scale without DIF items. OTA identified an 8-item WNSS (WNSS-8) which retained 67.2% of the test information of the original 21-item scale and had a Cronbach’s alpha of 0.83. It also showed significant correlations of 0.272 and −0.115 with the neuroticism and extraversion scales of Chinese NEO-Five Factor Inventory, respectively. Adequate model fit of the WNSS-8 was demonstrated by the confirmatory factor analysis. The Chinese WNSS-8 can be used to assess noise sensitivity without compromising reliability and validity.

## 1. Introduction

Noise, which refers to unwanted sounds, has become a substantial environmental problem around the world that impacts human health [1]. Noise may not only cause auditory problems, but may also have non-auditory effects on health [1]. Specifically, excessive noise exposure has been demonstrated to be associated with sleep problems, cognitive impairment, cardiovascular diseases, and some metabolic diseases [2,3,4]. However, individuals may experience different effects from noise due to their differences in sensitivity to noise. People who were more sensitive to noise would be more annoyed by noise or be more vulnerable to non-auditory health effects [5]. Hence, noise sensitivity may moderate the impact of noise on health. Indeed, it has been suggested that it is noise sensitivity that influences individual reactions to noise instead of noise exposure level [6]. Although it was assumed that people who were sensitive to noise were also sensitive to other environment issues such as odor [7], studies showed that noise sensitivity was different from other sensitivities [8]. For instance, neuroticism and smoking were demonstrated to be associated with noise sensitivity, while chemical sensitivity was in correlation with allergies and alcohol use [8]. Therefore, the independent assessment of noise sensitivity is necessary in epidemiological or interventional studies on the impact of noise on health.

The Weinstein Noise Sensitivity Scale (WNSS) is one of the most frequently used instruments for measuring noise sensitivity. Similar to other noise perception-related protocols, such as the International Organization for Standardization Technical Specifications report protocol which has undergone rigorous translations into 15 different languages [9], the WNSS has also been rigorously translated and tested in Swedish [10], German [11], Persian [12], Japanese [13], Italian [14], simplified Chinese [15], and traditional Chinese [16]. Despite the original WNSS being a 21-item unidimensional scale with each item rated on a 6-point Likert scale, multi-dimensional structures were identified in some translated versions. For instance, the Italian version showed two bipolar factors comprising the positively worded and the negatively worded items, respectively [14]. Moreover, a four-factor model was identified from the Persian version [12]. Nevertheless, the traditional Chinese version showed a unidimensional structure but had removed three items that did not fit well with the other items, resulting in an 18-item scale [16]. However, both the original 21-item version and the 18-item traditional Chinese might be too long to be incorporated in epidemiological studies. Therefore, a short form of only five items (short form of Weinstein Noise Sensitivity Scale; NSS-SF) was developed [17], which has been translated into Bulgarian and simplified Chinese [18,19]. However, the NSS-SF was derived from exploratory factor analysis (EFA), without thorough assessment of its adequacy when compared with the full 21-item version except for the total score correlation.

Classical test theory (CTT) and item response theory (IRT) are currently the two most popular methods for shortening scales. Under CTT, the observed score is assumed to be true with no errors in measurement, which is usually unrealistic [20]. Moreover, CTT focuses on assessment at the scale level, and establishes scale properties dependent on the sample. In contrast, IRT emphasizes the item level and establishes measurement properties independent of the sample [21]. Therefore, IRT has gained recent popularity. However, the selection of items remained subjective. Recently, the optimal test assembly (OTA) procedure was applied in patient-reported outcome measures (PROMs) for selecting the set of items that best resembles a collection of measurement properties of the full version [22]. Under specific constraints, e.g., number of items, it iteratively searches for the best set of items that optimize a specific objective, e.g., maximizing test information. Thus, OTA can optimize the attributes of a short test compared with the original test [23]. The OTA procedure has been shown to be able to produce reliable, replicable, and reproducible short versions with minimal length based on pre-specified and objective procedures [24]. 

To our knowledge, there is no short form of the traditional Chinese WNSS, and the current short forms of the WNSS have not been assessed by IRT or OTA. Therefore, this study aimed to obtain a short form of the traditional Chinese WNSS through an OTA procedure based on IRT, and to compare the performance of the obtained short form with the NSS-SF including reliability, validity, and test information. 

## 2. Materials and Methods

### 2.1. Participants

Chinese adults who were of ages 18 years or above who could understand Cantonese or could read and understand traditional Chinese were recruited. The eligible participants gave oral or written informed consent before taking the survey. Ethics approval for the study protocol was obtained from the institutional research committee.

Data of the participants were gathered from two studies. The first was a telephone survey in which the recruitment and survey were conducted via a telephone call by using random digit dialing, while the second was a household survey in which recruitment and survey were conducted via household visit.

IRT models usually require larger samples to acquire stable and accurate parameters. It was recommended that at least 500 respondents would be appropriate to conduct a 2-parameter model, such as the generalized partial credit model (GPCM), especially with more items [25].

### 2.2. Measurements

#### 2.2.1. The Weinstein Noise Sensitivity Scale (WNSS)

The original English version of the WNSS consists of 21 items. A 6-point response scale from agree strongly (1) to disagree strongly (6) is used. Negative-worded items were reversed before scoring which yield a higher score indicating higher sensitivity [5]. An 18-item traditional Chinese WNSS was obtained from the original English WNSS by standard forward–backward translation [16].

#### 2.2.2. The Chinese NEO-Five Factor Inventory (NEO-FFI)

The extraversion and neuroticism scales of the Chinese NEO-FFI were also administered [26]. Each scale comprises 12 items with each item being responded on a 5-point scale. Higher scores indicate higher level of extraversion and neuroticism for extraversion scale and neuroticism scale, respectively. The two scales were used for convergent validity according to previous findings [27].

### 2.3. Statistical Analysis

The telephone sample acted as the training sample based on which we conducted item selection. The household sample acted as the test sample based on which we assessed the performance of the short forms.

As the OTA is based on some IRT parameters, unidimensionality for the WNSS was firstly tested by minimum residual factoring of the polychoric correlation matrix exploratory factor analysis (EFA) to decide the usage of unidimensional IRT or multidimensional IRT [28]. Essential unidimensionality is acceptable if the first factor explained more than 20–40% of variance along with a value greater than 3 of the ratio of the eigenvalues for the first factor to the second factor [21]. 

Then, a GPCM was fitted to obtain the discrimination and difficulty parameters for each item. A higher discrimination indicates a greater ability of the corresponding item to differentiate respondents with different latent trait levels, while the difficulty parameter is specific to each pair of adjacent response categories of an item denoting the latent trait level with the same probability of choosing either response [21]. In our application, the latent trait level refers to the underlying noise sensitivity level. 

We assessed differential item functioning (DIF) by gender using the iterative Wald approach [29]. Specifically, the male and the female groups were taken as the focal and the reference groups, respectively. The Wald-2 approach (all-others-as-anchors model) was first conducted to identify the gender invariant items, which were the five items with the largest discrimination parameters (MaxA5 method). Then, the five items were set as the anchor items when using the Wald-1 approach to identify DIF items that showed a *p*-value < 0.05 [29,30]. 

After removing the items showing DIF, we obtained the non-DIF version of the WNSS for item selection by OTA. For each fixed test length, a short form was obtained by selecting a combination of items with the greatest total test information over the anchor points (−3, −1, 0, 1, 3) based on the GPCM by the branch-and-bound algorithm [24,31]. Total test information is the sum of all the item information, indicating the accuracy of estimating a latent trait level over the entire range of the trait level. A higher information means higher reliability [32]. Then, the optimal short form was taken as the minimal set of items that satisfied the three constraints: (1) maintained at least 95% of the Cronbach’s alpha of the non-DIF version; (2) the correlation of the summed score with that of the non-DIF version was at least 0.9; and (3) the correlation of the factor score with that of the non-DIF version was at least 0.9. The factor score of each participant was estimated based on GPCM by applying Bayes’ theorem, which was considered to give better estimates of the underlying latent trait levels [33].

With the training sample, the obtained short form of the WNSS was compared with the NSS-SF in terms of Cronbach’s alpha, the correlation between the summed scores, the correlation between the factor scores, convergent validity with the NEO-N/E, factorial validity and the test information. Furthermore, the Cronbach’s alpha, test information, and factorial validity were also compared between the obtained short form of the WNSS and the NSS-SF with the test sample. Factorial validity was assessed by conducting confirmatory factor analysis (CFA). The model fit of CFA model was considered adequate when the values of root mean square error of approximation (RMSEA), the standardized root mean square residual (SRMR), and the comparative fit index (CFI) were 0.08 or below, 0.08 or below, and 0.90 or higher, respectively [34].

The data analysis was conducted with RStudio 1.1.383. The EFA and DIF were performed with the R packages “psych” and “mirt”, respectively [35,36]. The package “ltm” and package “lpSolveAPI” were employed to run the OTA procedure [37,38]. The CFA model was performed with package “lavaan” [39].

## 3. Results

### 3.1. Participant Demographic Characteristics

A total of 1069 adults were recruited in two studies and the two samples shared similar sociodemographic characteristics. The training sample involved 569 Chinese adults with a mean age of 37 years (range: 18–91) who were recruited from the telephone survey. Of the sample, 63% were females. About half the sample had received bachelor or above education, while 8.8% received primary level education or below. The test sample involved 500 adults with an average age of 39 years (range: 18–88). The sample primarily consists of female (66%) participants, whereas only 5% received primary level education or below education.

### 3.2. Checking Unidimensionality of the 21-Item WNSS

The Kaiser-Meyer-Olkin statistic was 0.88 and Bartlett’s test was statistically significant (*p* < 0.001). The EFA showed that the ratio of the eigenvalues was greater than 3 (5.1 vs. 1.3). The first factor explained 24.1% of total variance, which was much higher than that of the second factor (6.1%). Therefore, essential unidimensionality of the WNSS was satisfied. However, EFA results revealed that the factor loading of Item 12 was negative, which should not be the case in the original WNSS. Hence, Item 12 was removed.

### 3.3. WNSS Item Properties and Selection

Table 1 shows the values of the discrimination parameters and item information for the 21 items of the WNSS. Discrimination for the 21 items ranged from 0.123 to 1.688. The five items with the highest discrimination parameters were: Item 10 (a = 1.688), Item 19 (a = 1.214), Item 18 (a = 1.206), Item 7 (a = 1.090), and Item 21 (a = 0.775). The Wald-2 test revealed DIF by gender only in Item 5 (*p* = 0.032). After setting the five items with the largest discrimination parameters as anchor items in Wald-1 test, Item 5 had a *p*-value of 0.013 for DIF, and thus it was also removed. Therefore, 19 items were retained after the iterative Wald test approach for performing OTA procedure. By OTA, the 8-item short form (WNSS-8), comprising items 6, 7, 10, 11, 13, 18, 19, 21, was the minimal set of items that satisfied the pre-specified criteria (traditional Chinese version: Appendix A). Table 2 shows the Cronbach’s alpha, correlation of the summed scores and factor scores of the WNSS-8 and the NSS-SF with those of the 19-item WNSS (WNSS-19). The Cronbach’s alpha and the correlation of summed scores of the NSS-SF did not meet the pre-specified criteria.

### 3.4. Testing the Short-Form Traditional Chinese WNSS

**Figure 1:**
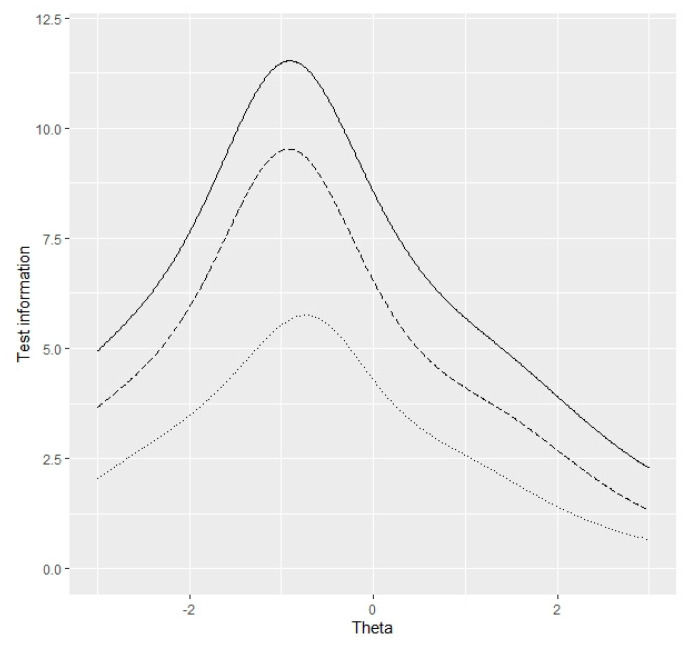
Test information curves of the original 21-item WNSS (solid curve), the WNSS-8 (longdash curve), and the NSS-SF (dotted curve).

The WNSS-8 and the NSS-SF showed similar convergent validity with the NEO-N/E (Table 3). Table 4 compares the one-factor CFA models of the WNSS-8 and the NSS-SF, and shows that both the WNSS-8 and the NSS-SF had a satisfactory model fit. Table 5 demonstrates the test information between the entire ability level and range (−3, 3) of different scales. The WNSS-8 and the NSS-SF kept 67.2% and 38.6% of the test information over the entire ability range, 73.1% and 43.2% over the ability range (−3, 3) of the original 21-item scale, respectively. Figure 1 shows the test information for the original 21-item WNSS the WNSS-8, and the NSS-SF between noise sensitivity level range of (−3, 3). The test information of the WNSS-8 more closely resembled that of the original 21-item WNSS than the NSS-SF. Item discrimination parameters for the WNSS-8 range from 0.587 to 1.775.

### 3.5. Testing the Short-Form Traditional Chinese WNSS with the Test Sample

Table 6 compares the Cronbach’s alpha, test information and factor validity of the one-factor CFA models of the WNSS-8 and the NSS-SF. Despite the fact that the two models demonstrated satisfactory model fit of CFA models, the NSS-SF showed a lower Cronbach’s alpha of 0.72. Moreover, the WNSS-8 and the NSS-SF kept 63.3% and 43.7%, respectively, of the test information over the entire ability range of the original 21-item version.

## 4. Discussion

This is the first study that used OTA methodology to obtain a short form of the WNSS for assessing noise sensitivity. The new WNSS-8 showed the best performance when considering internal consistency, correlation of summed scores, correlation of factor scores, convergent validity, construct validity and test information. 

The EFA revealed a negative factor loading for Item 12. As it is counter to the hypothesized direction of effect of the item, we decided to remove Item 12 from the OTA procedure. This should not greatly impact the results as the item information for Item 12 was the smallest, which means that Item 12 contributes the least for measuring the latent trait level [40]. The 18-item traditional Chinese version also has this item removed due to the small factor loading and communality [16]. Item 12 asked “It wouldn’t bother me to hear the sounds of everyday living from neighbors (footsteps, running water, etc.).” It was reported that only 6% of the residents in Hong Kong rated neighborhood noise as annoying compared with a percentage of 55% for traffic noise [41]. A previous study proposed that the apartment units in Hong Kong are usually separated by concrete walls and floors, and most people would not hear neighborhood noise such as the footsteps and running water [16]. Hence, people may react less to neighborhood noise and consider neighborhood noise not bothersome. Therefore, this item might not be applicable in a Hong Kong community setting. Moreover, the discrimination parameter for Item 12 was very low with a value of 0.123. This indicated that Item 12 might be unable to discriminate people with different levels of the latent trait [42]. In addition, the low information of the Item 12 indicated low precision and more measurement error of this item [25]. Hence, individuals with low trait level might score similarly or higher than those with high trait levels which induced the problematic performance of Item 12.

The iterative Wald test approach employed in this study has been demonstrated to reduce Type I and Type II errors [29]. The iterative Wald test approach identified the gender related DIF on Item 5 which asked “I am easily awakened by noise”. A previous study indicated that women had more awakenings and more awake time after sleep onset [43]. Therefore, women and men may not share the same norm in responding to this item even if they share similar sensitivity to noise. Of note, research on this aspect is quite limited, which calls for more studies investigating the role of gender on noise sensitivity. 

The convergent validity and construct validity of the WNSS-8 and the NSS-SF were similar. For reliability, a value of Cronbach’s alpha greater than 0.75 was suggested [44]. We set keeping 95% of the Cronbach’s alpha of the non-DIF version, which held a value of 0.81, as one of the rules for item selection. Using the training sample, the values of Cronbach’s alpha for the WNSS-8 and the NSS-SF were 0.83 and 0.67, respectively. Moreover, the Cronbach’s alpha for the NSS-SF with the test sample was 0.72. Hence, the Cronbach’s alpha of the NSS-SF was not adequate enough. Furthermore, the concurrent validity could be demonstrated by the scale scores’ correlation, which ranges from −1 to 1 [45]. A greater coefficient in absolute value indicates higher concurrent validity. The correlation of the summed scores of the WNSS-8 and the NSS-SF with the non-DIF version were 0.901 and 0.867, respectively. We proposed 0.90 as the criteria since a value greater than 0.90 indicates very high correlation [46]. Therefore, the concurrent validity of the NSS-SF was less adequate than that of WNSS-8.

The WNSS-8 and the NSS-SF retained 73.1% and 43.2% of the test information compared with the original 21-item version over the entire ability range, respectively, using the training sample. The removal of another three items induced 30% reduction of the test information. The comparison of the two short scales revealed similar results in the test sample. In view that higher test information represents higher accuracy of estimating the latent trait level, we proposed the WNSS-8 as a better short version [32]. Despite that there are no standard criteria for discrimination parameter, items with low discrimination, such as <0.4, were reported to have lower ability for differentiating the latent trait levels, carry smaller amounts of information, and are less able to reduce the estimation error [47]. The discrimination parameters for the items of the WNSS-8 ranged from 0.587 to 1.775, corresponding to moderate-to-high discrimination for assessment of noise sensitivity. Furthermore, the test information curves of the three scales demonstrated that the WNSS-8 more resembled the shape of the original full scale, which indicated that the WNSS-8 holds the similar ability for measuring noise sensitivity around the same latent trait level [48].

The result obtained from OTA, which uses the pre-specified criteria for conducting item selection, is replicable and reproducible [24]. We believe that OTA will show its value in shortening PROs for effective epidemiological research due to the burden caused by several and long PROs in surveys. However, there are also some limitations worth noting. The pre-specified criteria could be subjective to some extent. This study set 95% of the reliability and 90% of the correlation in view of the suggestive Cronbach’s alpha and a very high correlation indicated by a value greater than 0.9, which may highly resemble the original scale; other settings could be employed such as if the original Cronbach’s alpha was very high. Second, DIF by other characteristics such as age and responsiveness could be studied. Third, the convergent validity was low in this study. Testing convergent validity by the agreement with other noise sensitivity scales would be desirable in future studies.

## 5. Conclusions

The WNSS-8 could be used for assessing noise sensitivity with good reliability and validity. It allows more efficient assessment by healthcare professionals and researchers, especially in epidemiological studies with a battery of questionnaires.

## Figures and Tables

**Table 1:** WNSS items and discrimination parameters from the generalized partial credit model.

WNSS Items	Discrimination	Information
1. I wouldn’t mind living on a noisy street if the apartment I had was nice.	0.235	1.06
2. I am more aware of noise than I used to be.	0.329	1.55
3. No one should mind much if someone turns up his or her stereo full blast once in a while.	0.227	0.97
4. At movies, whispering and crinkling candy wrappers disturb me.	0.348	1.63
5. I am easily awakened by noise.	0.655	3.26
6 ^a^. If it’s noisy where I’m studying, I try to close the door or window or move someplace else.	0.832	4.15
7 ^a,b^. I get annoyed when my neighbors are noisy.	1.090	5.45
8 ^b^. I get used to most noises without much difficulty.	0.214	0.84
9. It would matter to me if an apartment I was interested in renting were located across from a fire station.	0.458	2.23
10 ^a^. Sometimes noises get on my nerves and get me irritated.	1.688	8.44
11 ^a^. Even music I normally like will bother me if I’m trying to concentrate.	0.605	3.00
12. It wouldn’t bother me to hear the sounds of everyday living from neighbors (footsteps, running water, etc.).	0.123	0.38
13 ^a^. When I want to be alone, it disturbs me to hear outside noises.	0.671	3.33
14. I’m good at concentrating no matter what is going on around me.	0.298	1.33
15. In a library, I don’t mind if people carry on a conversation if they do it quietly.	0.148	0.54
16. There are often times when I want complete silence.	0.345	1.62
17. Motorcycles ought to be required to have bigger mufflers.	0.539	2.64
18 ^a,b^. I find it hard to relax in a place that’s noisy.	1.206	6.03
19 ^a,b^. I get mad at people who make noise that keeps me from falling asleep or getting work done.	1.214	6.07
20. I wouldn’t mind living in an apartment with thin walls.	0.229	0.96
21 ^a,b^. I am sensitive to noise.	0.775	3.86

WNSS: Weinstein Noise Sensitivity Scale; ^a^: items of the 8-item WNSS; ^b^: items of the NSS-SF.

**Table 2:** Properties of the WNSS-19, WNSS-8 and NSS-SF.

Short-Form Length	Cronbach’s Alpha (95% CI)	Correlation of Summed Scores (95% CI)	Correlation of Factor Scores (95% CI)
WNSS-19	0.81 (0.79, 0.83)	1.000 (1.000, 1.000)	1.000 (1.000, 1.000)
WNSS-8	0.83 (0.81, 0.85)	0.901 (0.884, 0.915)	0.982 (0.979, 0.985)
NSS-SF	0.67 (0.62, 0.71)	0.867 (0.826, 0.909)	0.904 (0.869, 0.940)

WNSS-19: 19-item Weinstein Noise Sensitivity Scale; WNSS-8: 8-item Weinstein Noise Sensitivity Scale; NSS-SF: short form of Weinstein Noise Sensitivity Scale.

**Table 3:** Convergent validity of the WNSS-21, WNSS-8 and NSS-SF with extraversion and neuroticism scales of Chinese NEO-FFI.

Measures	WNSS-21 (95% CI)	WNSS-8 (95% CI)	NSS-SF (95% CI)
NEO-N	0.294 (0.215, 0.374)	0.272 (0.192, 0.352)	0.269 (0.189, 0.349)
NEO-E	−0.112 (−0.200, −0.035)	−0.115 (−0.197, −0.032)	−0.118 (−0.200, −0.035)

NEO-FFI: NEO-Five Factor Inventory; WNSS-21: 21-item Weinstein Noise Sensitivity Scale; WNSS-8: 8-item Weinstein Noise Sensitivity Scale; NSS-SF: short form of Weinstein Noise Sensitivity Scale.

**Table 4:** Comparison of the CFA models of the WNSS-8 and NSS-SF.

Short Versions	χ^2^	RMSEA (90% CI)	SRMR	CFI
WNSS-8	61.477	0.069 (0.050, 0.090)	0.038	0.957
NSS-SF	8.852	0.040 (0.000, 0.083)	0.024	0.990

CFA: confirmatory factor analysis; WNSS-8: 8-item Weinstein Noise Sensitivity Scale; NSS-SF: short form of Weinstein Noise Sensitivity Scale; RMSEA: root mean square error of approximation; SRMR: standardized root mean square residual; CFI: comparative fit index.

**Table 5:** Test information of the WNSS-21, WNSS-8 and NSS-SF.

Index	WNSS-21	WNSS-8	NSS-SF
Test information	59.36	39.89 (67.2%)	22.93 (38.6%)
Test information (−3, 3)	43.42	31.72 (73.1%)	18.75 (43.2%)

WNSS-21: 21-item Weinstein Noise Sensitivity Scale; WNSS-8: 8-item Weinstein Noise Sensitivity Scale; NSS-SF: short form of Weinstein Noise Sensitivity Scale.

**Table 6:** Comparison of reliability and validity of WNSS-8 and NSS-SF with test sample.

Short Versions	Cronbach’s Alpha	Test Information	Factorial Validity			
			χ^2^	RMSEA (90% CI)	SRMR	CFI
WNSS-8	0.80 (0.78, 0.83)	40.01 (63.3%)	73.229	0.073 (0.058, 0.089)	0.049	0.923
NSS-SF	0.72 (0.68, 0.76)	27.62 (43.7%)	8.340	0.037 (0.000, 0.076)	0.022	0.991

WNSS-8: 8-item Weinstein Noise Sensitivity Scale; NSS-SF: short form of Weinstein Noise Sensitivity Scale; RMSEA: root mean square error of approximation; SRMR: standardized root mean square residual; CFI: comparative fit index.

## Data Availability

The data are not publicly available due to no agreement from the study participants for open sharing of the dataset.

## Appendix A

**Table:**

The Traditional Chinese Version of the WNSS-8						
	非常同意					非常 不同意
1. 如我研習的地方嘈吵，我會嘗試關門、關窗或移至別的地方。	☐_6_	☐_5_	☐_4_	☐_3_	☐_2_	☐_1_
2. 當我的鄰居嘈吵時，我會覺得煩擾。	☐_6_	☐_5_	☐_4_	☐_3_	☐_2_	☐_1_
3. 有時候，噪音會令我心煩及生氣。	☐_6_	☐_5_	☐_4_	☐_3_	☐_2_	☐_1_
4. 當我專心時，即使平常我喜歡的音樂也會覺得煩擾。	☐_6_	☐_5_	☐_4_	☐_3_	☐_2_	☐_1_
5. 當我想獨處時，外來的聲音會打擾我。	☐_6_	☐_5_	☐_4_	☐_3_	☐_2_	☐_1_
6. 我很難在一個嘈雜的地方放鬆。	☐_6_	☐_5_	☐_4_	☐_3_	☐_2_	☐_1_
7. 我惱怒那些製做噪音，令我不能入睡或完成工作的人。	☐_6_	☐_5_	☐_4_	☐_3_	☐_2_	☐_1_
8. 我對噪音很敏感。	☐_6_	☐_5_	☐_4_	☐_3_	☐_2_	☐_1_
